# Effect of Clinical and Genetic Factors on the Development of Postoperative Atrial Fibrillation After Coronary Artery Bypass Grafting (CABG) in Egyptian Patients Receiving Beta-Blockers

**DOI:** 10.1007/s10557-022-07380-6

**Published:** 2022-09-15

**Authors:** Dina M. K. El Gindy, Mohamed H. Solayman, Ramy Khorshid, Mona F. Schaalan, Lamia M. El Wakeel

**Affiliations:** 1 Department of Clinical Pharmacy and Pharmacy Practice, Faculty of Pharmacy, Misr International University, Cairo, Egypt; 2 Department of Clinical Pharmacy and Pharmacy Practice, Faculty of Pharmacy, Ain Shams University, Cairo, Egypt; 3 Clinical Pharmacy Unit, Faculty of Pharmacy and Biotechnology, German University in Cairo, Cairo, Egypt; 4 Department of Cardiovascular and Thoracic Surgery, Ain Shams University Hospital, Faculty of Medicine, Ain Shams University, Cairo, Egypt

**Keywords:** Beta-1 adrenergic receptor, G protein-coupled receptor kinase, Gene polymorphism, Beta-blocker, Postoperative atrial fibrillation, Coronary artery bypass grafting

## Abstract

**Purpose:**

Prophylactic beta-blockers are recommended to prevent postoperative atrial fibrillation (POAF) after coronary artery bypass grafting (CABG). Polymorphisms in the beta-1 adrenergic receptor (ADRB1) and G protein-coupled receptor kinase 5 (GRK5) genes are associated with variable responses to beta-blockers. The aim of this study was to determine the clinical and genetic factors that influence the response to beta-blockers for POAF prophylaxis after CABG.

**Methods:**

Patients undergoing isolated CABG and receiving prophylactic beta-blockers (*n* = 249) were prospectively recruited and followed up for 6 postoperative days. Genotyping of ADRB1 rs1801253, and 3 GRK5 SNPs (rs3740563, rs10787959, and rs17098707) was performed.

**Results:**

Of the 249 patients, 52 patients (20.8%) experienced POAF. Age, hypertension, vasopressor use, calculated POAF risk score, GRK5 rs2230345 T-allele, and GRK5 rs3740563 A-allele were associated with POAF despite beta-blocker prophylaxis. The multivariate analysis revealed that age [odds ratio (OR) 1.06, 95% CI 1.02–1.11, *p* = 0.003] and GRK5 rs2230345 T-allele [OR 2.81, 95% CI 1.39–5.67, *p* = 0.004] were independent predictors of POAF after CABG despite beta-blocker prophylaxis.

**Conclusion:**

GRK5 rs2230345 T-allele carriers were less responsive than AA genotype carriers to prophylactic beta-blockers for the prevention of POAF after CABG.

The study was registered on http://clinicaltrials.gov in March 2019, with trial registration number (TRN): NCT03871647.

## Introduction

Postoperative atrial fibrillation (POAF) is a common complication that affects 20–40% of patients after isolated coronary artery bypass grafting (CABG), 40–50% of patients after valve surgery, and up to 60% of people after combined valvular and CABG surgery [1]. POAF has various negative outcomes including a higher risk of infection, respiratory or renal failure, cardiac arrest, and stroke compared with CABG patients who remain in sinus rhythm after surgery. Consequently, POAF has been associated with longer ICU and hospital stay, increased in-hospital mortality, and higher hospital costs [2].

Several clinical factors have been found to increase the risk of POAF such as older age, co-morbid diseases including hypertension, chronic obstructive pulmonary disease, and atrial fibrillation, withdrawal of beta-blocker medications in patients using them chronically, and the type of procedure performed [3]. In addition, genome-wide association studies have identified several genetic variants that are associated with POAF and could play a role in its etiology [4, 5].

The main cause of POAF pathogenesis is sympathetic adrenergic stimulation. Thus, various European and American published guidelines recommend the use of perioperative beta-blockers for POAF prophylaxis [6, 7] as they decrease the sympathetic tone leading to prolonged atrial refractoriness and decreased initiation of arrhythmias [8, 9].

Despite the use of beta-blockers, approximately 20% of patients develop POAF following CABG surgery [10]. Several studies suggested that the variability in the response to beta-blockers could be attributed to polymorphism in the genes encoding for the β1-adrenergic receptor (ADRB1), and a kinase that terminates its signaling known as G protein-coupled receptor kinase 5 (GRK5) [10–12].

Notably, prediction tools that identify patients who will benefit from prophylactic beta-blockers for the prevention of POAF after cardiac surgery are not common in clinical practice. Some researchers have proposed that there is a reluctance to expose patients to the potential adverse effects of other alternative prophylactic medications such as antiarrhythmic medications [13]. Accordingly, the current study aimed to assess the impact of clinical and genetic factors that may influence the response to beta-blockers required for POAF prophylaxis after CABG. This current research will aid in the personalization of therapy by identifying patients who will benefit the most from perioperative beta-blockers and those who will opt for other prophylactic medications. Consequently, the risk of development of POAF will be reduced.

## Methods

### Patients and Sample Collection

This prospective observational cohort study was performed on 256 patients admitted for isolated CABG surgery. Patients were consecutively recruited from the Academy of Cardiovascular & Thoracic Surgery-Ain Shams University, Cairo, Egypt, between March 2019 and December 2020. Patients were included in this study if their age was above 18 years, were candidates for isolated CABG surgery without concurrent valve surgery, and were receiving prophylactic beta-blockers. Prophylactic use of beta-blockers was defined as the use of beta-blockers during the hospitalization period of CABG, provided that those with POAF would have received the beta-blockers prior to the occurrence of AF. Patients were excluded from this study if they had uncontrolled thyroid disease, recent treatment with amiodarone before the occurrence of POAF, a serum creatinine > 2 mg/dL before the surgery, or the occurrence of persistent or permanent AF, atrial flutter, or atrial tachycardia on electrocardiogram (ECG) on the day of operation. A written informed consent was obtained from each participant preoperatively prior to recruitment in the study. The study was conducted in accordance to the guidelines of the declaration of Helsinki and its updates in 2013. The study protocol was approved by the local Ethics Committee Review Board at Faculty of Pharmacy, Ain Shams University, Cairo, Egypt (No: PhD 53). The study was registered at http://clinicaltrials.gov, with identifier number: NCT03871647.

All patients underwent isolated CABG procedures and received standard postoperative care according to the institution’s standard protocol. Data collected for all patients included demographics, comorbid diseases, and preoperative and postoperative medications. For each patient, the following intraoperative characteristics were reported including the number of grafts, bypass time, and cross-clamp time, as well as the use of intra- or postoperative vasopressors. All data were collected in a standardized data collection sheet and postoperative AF risk score was calculated [3]. For the calculation of the postoperative AF risk score, the following variables were considered: age, history of atrial fibrillation, history of chronic obstructive pulmonary disease, concurrent valve surgery, withdrawal of beta-blockers, withdrawal of ACEI, and the use of the following medications: preoperative and postoperative beta-blockers, preoperative and postoperative ACEI, postoperative beta-blockers, postoperative potassium supplementation, and non-steroidal anti-inflammatory drugs. A risk score < 14 indicated low risk; a score of 14–31 indicated medium risk; and a score > 31 indicated high risk. Patients underwent continuous 24–h ECG monitoring for the first 6 days. Diagnosis of new-onset POAF was based on the continuous postoperative ECG monitoring. Atrial fibrillation (AF) diagnostic criteria included the absence of discrete P waves, an RR interval following a non-repetitive form, and the ranges of ventricular rate usually ranging between 90 and 170 beats/min. AF that occurred at any time during the first six postoperative days for at least 5 min or led to a change in treatment (such as drug administration or cardioversion) despite electrolyte correction was being considered as a qualifying event. The POAF was identified by physicians or trained nursing staff using ECG recording obtained and an experienced cardiologist was available on a daily basis for rhythm-related consultations.

### Genotyping/Pharmacogenetics Analysis

Whole peripheral blood samples (3 ml) of patients planned to undergo CABG were withdrawn into an ethylenediaminetetraacetic acid-containing tube before surgery and stored at − 80 °C until DNA was extracted using QIAamp DNA Blood Mini Kit (Qiagen, Hilden, Germany) according to the manufacture’s guidelines. Genotyping for genetic analysis was done using TaqMan® genotyping master mix and TaqMan® single nucleotide polymorphism (SNP) Genotyping Assay (Applied Biosystems, Foster City, CA, USA) for the 4 SNPs assayed in this study; ADRB1 rs1801253, and 3 GRK5 SNPs (rs3740563, rs10787959, and rs17098707). The assay was performed using Qiagen’s real-time PCR cycler (Rotor- gene Q®) (Qiagen, Hilden, Germany) by an investigator blinded to the study.

### Statistical Analysis

Continuous variables were presented as means ± SDs or median (interquartile range) whereas categorical variables were presented as frequency and percentages. Either independent-sample *t*-test or Mann–Whitney test was used to analyze continuous variables as appropriate while categorical variables were analyzed using the Chi-square or Fisher exact test as appropriate. The Chi-square test was used to test the deviation of genotype distribution from the predicted genotype frequencies based on the Hardy–Weinberg equilibrium. The frequency of the variant alleles of the studied SNPs was reported in Egyptians and compared with other populations. ADRB1 and GRK5 genotypes were analyzed using the dominant genetic model (i.e., wild-type homozygotes versus one or two variant allele carriers) due to low minor allele frequencies (MAF). Variables significantly associated with POAF in the univariate analysis were analyzed using stepwise binary logistic regression analysis. Only variables significant in the multivariable model were entered into the final model. All tests were two-sided, and *p*-value < 0.05 was considered statistically significant. Given our sample size, the minor allele frequencies of ADRB1 rs1801253, GRK5 rs2230345, GRK5 rs3740563, and GRK5 rs10787959, and an a priori expected POAF prevalence of 20%, we estimated that we had 80% power to detect an odds ratio = 2.72 for ADRB1 rs1801253, odds ratio = 2.75 for both GRK5 rs2230345 and GRK5 rs3740563, and odds ratio = 2.47 for GRK5 rs10787959 for the binary outcome in univariate analysis. Statistical analysis was performed using SPSS statistical software package version 16.0 (SPSS, Inc, Chicago, IL). Figures were created using Graphpad prism version 5 (San Diego, CA, USA).

## Results

**Fig. 1:**
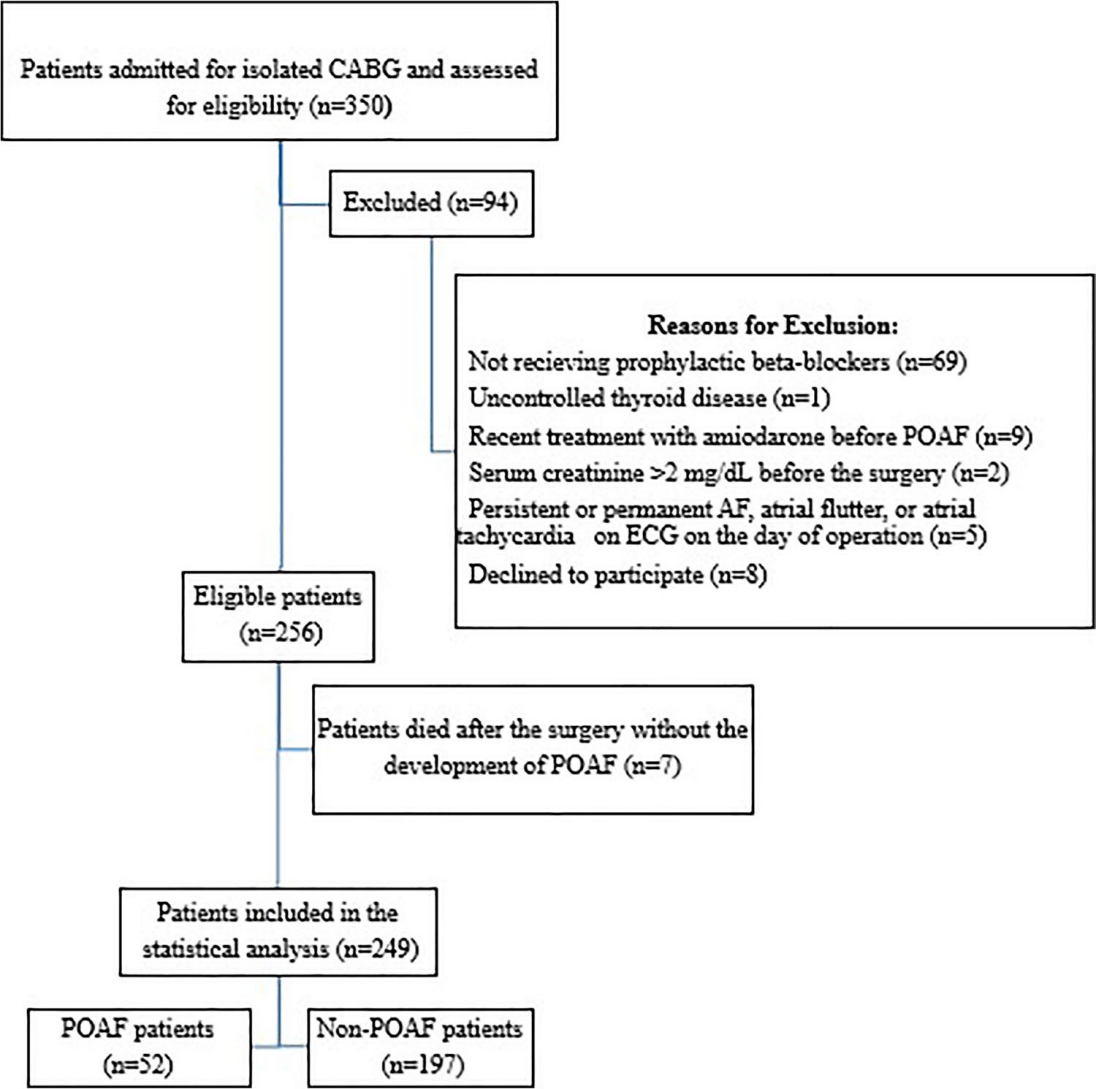
Flow diagram of the cohort study

**Fig. 2:**
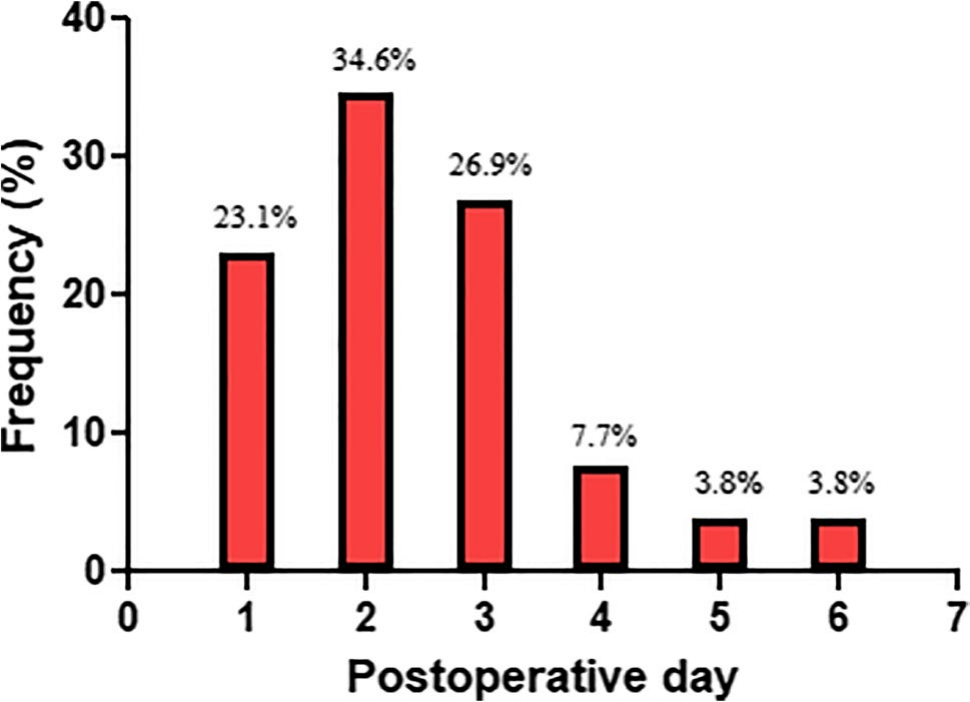
Day of the initial occurrence of POAF episode

Out of a total of 256 patients enrolled, 7 patients were excluded from the study due to death after the surgery without the occurrence of POAF and only 249 completed the study as presented in (Fig. 1). The demographics and clinical characteristics of the enrolled patients stratified according to the presence or absence of POAF are summarized in (Table 1). Males represented 81.1% of the total cohort. The mean age of the studied cohort was 57.3 ± 8.6 (years). Fifty-two participants (20.8%) suffered from POAF after CABG despite prophylactic beta-blockers. Most of the POAF episodes occurred on the second postoperative day as shown in (Fig. 2). From the results of the univariate analysis, several clinical factors were associated with the occurrence of POAF and included age, hypertension, use of vasopressor medications, and the calculated POAF risk index. Other clinical factors were comparable between the 2 groups. Patients who developed postoperative atrial fibrillation were significantly older than those who did not experience POAF (*p* = 0.002). The percentage of hypertensive patients was statistically higher in the POAF group compared with the non-POAF group (*p* = 0.038). Also, the use of vasopressors such as (dopamine, dobutamine, norepinephrine, epinephrine) was statistically higher in the POAF group compared with the non-POAF group (*p* = 0.034). The POAF group had a higher calculated postoperative AF risk score compared with the non-POAF group (*p* = 0.030).

**Table 1:** Demographics and clinical characteristics of the enrolled patients

Variable	Non-POAF (*n* = 197)	POAF (*n* = 52)	*P*-value
Age in years; mean ± SD	56.5 ± 8.8	60.6 ± 6.8	^ǂ^0.002*
Gender; *n* (%)			
* Males*	160 (81.2%)	42 (80.8%)	
* Females*	37 (18.8%)	10 (19.2%)	^#^0.941
Smokers; *n* (%)	83 (42.1%)	25 (48.1%)	^#^0.442
Weight in kg; mean ± SD	86.0 ± 14.5	87.3 ± 13.2	^ǂ^0.561
Height in cm; mean ± SD	166.9 ± 8.6	164.9 ± 7.8	^ǂ^0.131
BMI in kg/m^2^; mean ± SD	30.9 ± 4.9	32.2 ± 5.2	^ǂ^0.097
Co-morbid diseases; *n* (%)			
Diabetes mellitus	112 (56.9%)	30(57.7%)	^#^0.913
COPD	9 (4.6%)	0 (0%)	^##^0.211
Hypertension	121 (61.4%)	40 (76.9%)	^#^0.038*
Liver disease	23 (11.7%)	5 (9.6%)	^#^0.676
LVEF < 50	37 (18.8%)	13 (25%)	^#^0.319
Preoperative medications; *n* (%)			
Statins	128 (65%)	36 (69.2%)	^#^0.565
Trimetazidine	63 (32%)	17 (32.7%)	^#^0.922
Ivabradine	13 (6.6%)	2 (3.8%)	^##^0.743
ACEI/ARBs	117 (59.4%)	30 (57.7%)	^#^0.825
Insulin	59 ( 29.9%)	17 (32.7%)	^#^0.702
Oral hypoglycemics	75(38.1%)	20 (38.5%)	^#^0.959
Calcium channel blockers	17 (8.6%)	5 (9.6%)	^##^0.787
Intraoperative characteristics			
no of grafts; *n* (%)			
1–2	68 (34.5%)	23 (44.2%)	^#^0.180
3	101(51.3%)	26(50%)	
4–5	28 (14.2%)	3 (5.8%)	
BPT in min.; mean ± SD	106.2 ± 31.6	103.4 ± 36.0	^ǂ^0.586
CCT in min.; mean ± SD	63.8 ± 22.0	76.0 ± 119.9	^ǂ^0.474
Intra- or postoperative use of vasopressor; *n* (%)	157 (79.7%)	48 (92.3%)	^#^0.034*
Post-operative medications; *n* (%)			
ACEI/ARBs	51 (25.9%)	17 (32.7%)	^#^0.327
Calcium channel blockers	18 (9.1%)	9 (17.3%)	^#^0.092
NSAIDs	28 (14.2%)	7 (13.5%)	^#^0.890
Potassium supplementation	188 (95.4%)	48 (92.3%)	^##^0.480
Antipsychotics	4 (2%)	4(7.7%)	^##^0.062
Ivabradine	102 (51.8%)	24 (46.2%)	^#^0.471
Postoperative AF risk score (median [IQR])	2 (-4–7)	6 (1–12)	^$^0.030*

Statistical significances are derived by the Chi−square test (#) /Fisher exact test (##) analysis for categorical/dichotomous variables as appropriate or independent−sample t−test (ǂ) or Mann–Whitney test ($) for continuous variables.*p < 0.05

*ACE−I* angiotensin−converting enzyme inhibitors, *AF* atrial fibrillation, *ARBs* angiotensin II receptor blockers, *BPT* bypass time, *BMI* body mass index, *CCT* cross−clamp time, *COPD* chronic obstructive pulmonary disease, *LVEF* left ventricular ejection fraction, *NSAIDs* non−steroidal anti−inflammatory drugs, *POAF* postoperative atrial fibrillation

### Genotype Frequencies

The genotypic frequencies of the 4 studied SNPs (rs2230345, rs3740563, rs1801253, and rs10787959) in the total cohort and in both the POAF and non-POAF subgroups are shown in (Table 2). The observed allele frequencies did not deviate from the Hardy–Weinberg equilibrium expectations in the total cohort, in the POAF and non-POAF subgroups.

**Table 2:** The genotypic frequencies of the 4 studied SNPs

SNP	Total (*n* = 249)	*p*-value	Non-POAF (*n* = 197)	*p*-value	POAF (*n* = 52)	*p*-value
rs2230345; *n* (%)						
AA	200 (80.3%)	0.85	166 (84.3%)	0.77	34 (65.4%)	0.95
AT	46 (18.5%)		30 (15.2%)		16 (30.8%)	
TT	3 (1.2%)		1 (0.5%)		2 (3.8%)	
rs3740563; *n* (%)						
CC	203 (81.5%)	0.10	168 (85.3%)	0.10	35 (67.3%)	0.81
AC	41 (16.5%)		26 (13.2%)		15 ( 28.8%)	
AA	5 (2%)		3 (1.5%)		2 (3.8%)	
rs1801253; *n* (%)						
CC	87 (34.9%)	0.80	69 (35%)	0.89	18 (34.6%)	0.78
CG	122 (49%)		96 (48.7%)		26 (50%)	
GG	40 (16.1%)		32 (16.2%)		8 (15.4%)	
rs10787959; *n* (%)						
GG	120 (48.2%)	0.95	97 (49.2%)	0.42	23 (44.2)	0.08
AG	106 (42.6%)		79 (40.1%)		27 (51.9%)	
AA	23 (9.2%)		21 (10.7%)		2 (3.8%)	

***p**** p*−value when using the χ2 test to test the deviation of genotype distribution from the predicted genotype frequencies based on the Hardy–Weinberg equilibrium

*SNP* single nucleotide polymorphism, *POAF* postoperative atrial fibrillation

**Fig. 3:**
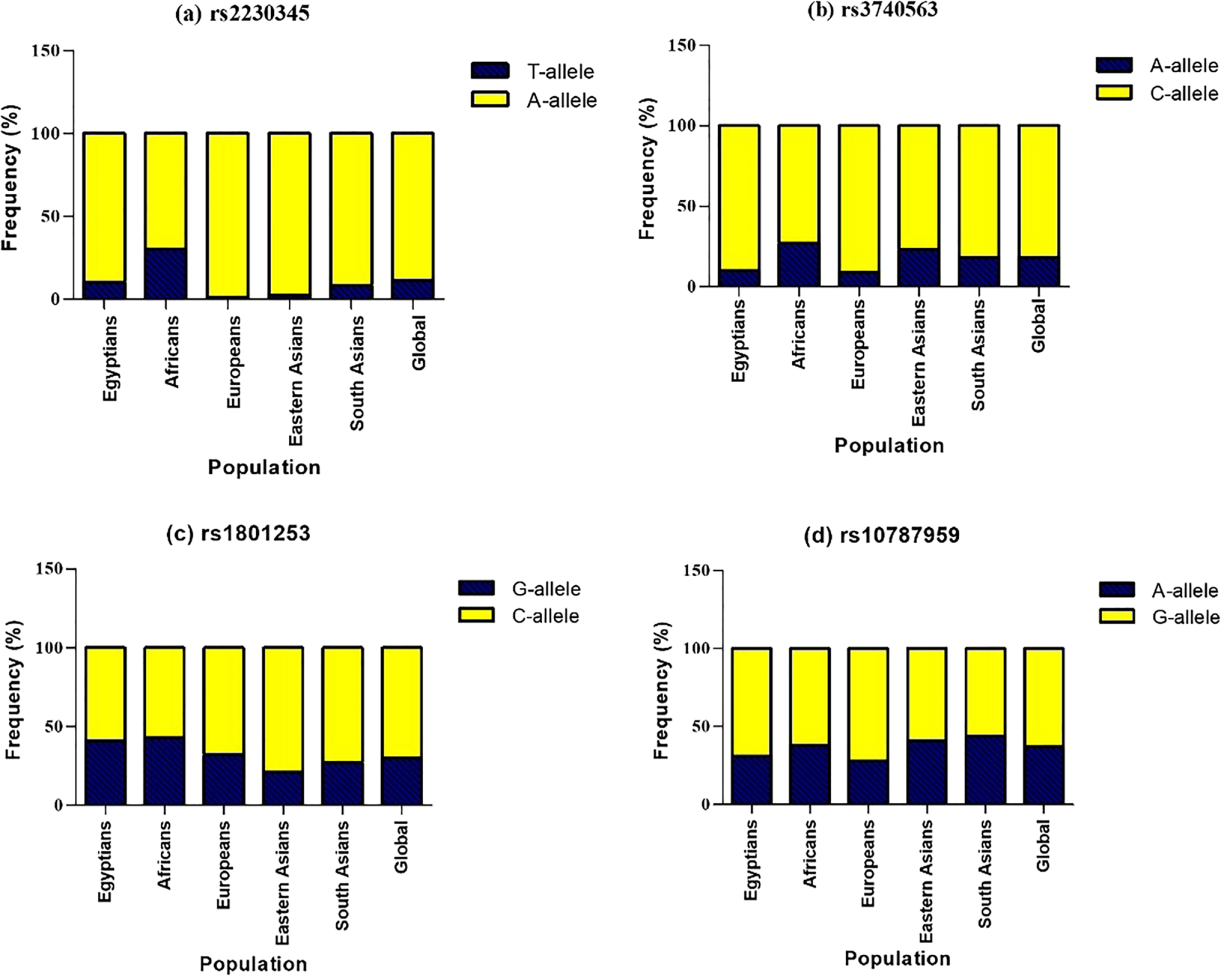
Comparison of allele frequencies of the studied SNPs in Egyptians compared with other populations. **a** GRK5 rs2230345 (yellow bars: A-allele, blue bars: T-allele), **b** GRK5 rs3740563 (yellow bars: C-allele, blue bars: A-allele), **c** ADRB1 rs1801253 (yellow bars: C-allele, blue bars: G-allele), and **d** GRK5 rs10787959 (yellow bars: G-allele, blue bars: A-allele). Allele frequencies obtained from the 1000 Genome, phase 3 data [22]

Comparison of the MAF of the studied SNPs in Egyptians with those reported in African, European, Asian, and the global population showed different MAF patterns between Egyptians and other races, as shown in (Fig. 3). In Egyptians, the rs2230345 T-allele was close to the south Asians but significantly higher than Europeans and Eastern Asians and lower than Africans while the frequencies of the rs3740563 A-allele and the rs10787959 A-allele were almost similar to Europeans but lower than the other populations. The rs1801253 G-allele in Egyptians was similar to Africans, lower than Europeans but higher than Asians.

When assessing the 4 SNPs (rs2230345, rs3740563, rs1801253, and rs10787959) under a dominant model of inheritance (Table 3), it was found that for the rs2230345 SNP, Egyptian patients with AT or TT genotypes were 2.84 more likely to develop POAF after CABG (OR = 2.84, 95% CI:1.43–5.64, *p* = 0.002) compared with patients with the AA genotype despite prophylactic beta-blockers. In addition, Egyptian patients with the rs3740563 SNP polymorphism (AC or AA) were associated with a 2.81-fold increased risk of developing POAF after CABG (OR = 2.81, 95% CI: 1.40–5.67, *p* = 0.003) compared with patients with the homozygous genotype (CC).

**Table 3:** Dominant model of inheritance

SNP	Non-POAF (*n* = 197)	POAF (*n* = 52)	Univariate OR (95% CI)	*P*-value
rs2230345; *n* (%)				
AA	166 (84.3%)	34 (65.4%)	2.84 (1.43–5.64)	0.002*
AT + TT	31(15.7%)	18 (34.6%)		
rs3740563; *n* (%)				
CC	168 (85.3%)	35 (67.3%)	2.81(1.40–5.67)	0.003*
CA + AA	29 (14.7%)	17 (32.7%)		
rs1801253; *n* (%)				
CC	69 (35.0%)	18 (34.6%)	1.02 (0.54–1.94)	
CG + GG	128 (65.0%)	34 (65.4%)		0.956
rs10787959; *n* (%)				
GG	97 (49.2%)	23 (44.2%)		
AG + AA	100 (50.8%)	29 (55.8%)	1.22 (0.66–2.26)	0.520

*CI* confidence interval, *POAF* postoperative atrial fibrillation, *OR *odds ratio, *SNP* single nucleotide polymorphism

^*^p < 0.05

### Risk Prediction Model for POAF

The assessment of the multivariate stepwise binary regression analysis was performed and included age, hypertension, postoperative AF risk score, use of vasopressors, rs2230345 SNP assessed under the dominant model (TT / AT vs AA), and rs3740563 SNP assessed under the dominant model (AA / CA vs CC). The results showed that only increasing age and presence of rs2230345 T-allele were independent predictors of postoperative atrial fibrillation in patients receiving prophylactic beta-blockers after controlling the effect of the other variables. The final model that is composed of both age and rs2230345 genotype (*p* = 0.003 and *p* = 0.004, respectively), shown in (Table 4), could explain 11% (Nagelkerke *R*^2^) of the variability in the binary outcome.

**Table 4:** The final model

Variable	OR	95%CI		*P*-value
		Lower	Upper	
rs2230345 (T-allele)	2.81	1.39	5.67	0.004*
Age (years)	1.06	1.02	1.11	0.003*

*OR* multivariate odds ratio; *CI* confidence interval

^*^p < 0.05

## Discussion

This study is the first observational study to address the clinical and genetic markers affecting the response to prophylactic beta-blockers required for the prevention of POAF after CABG in the Egyptian population.

In this study, POAF was reported in 20.8% of the patients despite the use of beta-blockers for POAF prophylaxis after CABG which is close to that reported by Kertai et al. in their discovery cohort (19.7%) and in their replication cohort (17.1%) [10].

We found that increasing age was the most prominent clinical risk factor for POAF despite prophylaxis with beta-blockers. Notably, aging causes cardiac fiber loss, increased fibrosis, and collagen deposition in the atria, mainly at the sinoatrial node, altering atrial electrical characteristics [2, 14].

In addition, our results showed that the presence of GRK5 SNP rs2230345 T-allele, known as GRK5-Leu41, was independently associated with higher POAF occurrence compared with homozygous Gln41 in CABG patients receiving prophylactic beta-blockers; thus, GRK5 Gln41Leu different genotypes could be used as a genetic marker for the prediction of the response to beta-blockers in POAF prophylaxis. The frequency of the rs2230345 T-allele in Egyptians was found to be 10% which is close to the frequency of south Asians (8%) but lower than Africans (30%) and higher than Europeans (1%) and Eastern Asians (2%).

G protein-coupled receptor kinase 5 is highly expressed in the normal human heart and is encoded by the GRK5 gene. GRK5 regulates catecholamines, which bind and activate adrenergic receptors [15]. The genetic polymorphism in GRK5 SNP rs2230345 (GRK5 122 A > T; Gln41Leu) in which Leucine (Leu) is substituted for glutamine (Gln) causes a gain-of-function for the G-protein receptor kinase. Thus, it leads to enhanced loss-of-function (desensitization) of beta-1 adrenergic receptor (β1AR) during persistent agonist exposure [16, 17] mimicking the effect of beta-blockers in genetic mouse models [17]. The effect of this polymorphism has not been studied before in association with the response to beta-blockers required for POAF prophylaxis in patients undergoing CABG. Nevertheless, studies on patients with heart failure or hypertension yielded conflicting results [11, 18, 19]. In accordance with our findings, a prospective cohort study that included Caucasian and African American heart failure patients reported a detrimental effect on survival in GRK5-Leu41 carriers treated with beta-blockers in Caucasians and among the whole cohort. However, in African American patients, there was no association between GRK5-Gln41Leu polymorphism genotypes and heart failure outcomes [11]. In another cohort of hypertensive patients on beta-blockers, there was no significant difference in the cardiac adverse outcomes (first occurrence of death, nonfatal MI, or stroke) between patients with homozygote Gln-41 or Leu-41 variant. The lack of pharmacogenetic interaction in that study was supported by blood pressure response data, which showed that both Leu41-carriers and Gln41-homozygote-carriers had similar responses to atenolol [18]. In contrast to our findings, a study by Ramalingam et al. on Indian heart failure patients found that patients carrying GRK5 Leu41 variants and receiving beta-blocker therapy showed better ejection fraction and increased hospitalization-free survival compared with patients with wild-type genotype on beta-blockers [19]. The discrepancy between our results and those of Ramalingam et al. [19] could be attributed to several reasons. First, Ramalingham et al. reported a significant reduction in the beta-blocker (carvedilol) dose in the GRK5 Leu41 carriers from the initial dose to the final one. Yet, there was no change in the doses of the GRK4 Gln-41 carriers from baseline to the end of the study. Second, the authors did not clarify the other medications administered by the GRK5 Leu41 and GRK5 Gln41 carriers specifically and whether they were similar or not. Hence, this could raise the doubt if the beneficial effects observed in the GRK5 Leu-41 carriers were all attributed to the effect of beta-blockers or other medications as well.

In the current study, another genetic polymorphism in the GRK5 gene (rs3740563; A > C) was studied in association with the development of POAF. Our results revealed that patients with AC or AA variants had a higher risk for the development of POAF despite prophylactic beta-blockers; however, this SNP had a limited role in predicting the response to beta-blockers in POAF prophylaxis. The frequency of the A-allele in Egyptians was 10% which is almost similar to Europeans (9%) but lower than the other populations. Similarly, Kertai et al. found that the risk allele “A” of rs3740563 was significantly associated with a higher risk of POAF despite perioperative beta-blocker prophylaxis. Even though the potential mechanism of action of this SNP was not studied by Kertai et al., the authors postulated that it might be mediated via DNA transcription regulation [10]. Although Li et al. did not study the effect of this SNP on the response to beta-blockers, they found that each additional copy of minor allele A in Asian patients was associated with a 1.31-fold increased risk of development of POAF in the discovery cohort (OR = 1.31; 95% CI: 1.09–1.58, *p*-value = 0.005) but it was replicated in the validation cohort without significance [20].

The third GRK5 SNP evaluated was rs10787959 (A > G). The frequency of the rs10787959 A-allele in Egyptians was 31% which is almost similar to Europeans (28%) but lower than the other populations. In the current study, there was no association between this SNP and POAF development in beta-blockers-users. In alignment with our study, Liu et al. could not find an association between this SNP and POAF development in the validation study that was done on Asians regardless of beta-blocker use [20]. In contrast to our results, Kertai et al. found a significant association between the A-variant of the rs10787959 SNP and POAF despite perioperative beta-blocker use [10]. The variation between these two studies could be attributed to difference in sample sizes, patient’s comorbidities, and medications administered between the studies.

It is worth mentioning that only one published study so far has studied the effect of ADRB1 rs1801253 (Arg389Gly) polymorphism on the development of POAF following cardiac surgeries. Jeff et al. found that those with the ADRB1 Gly389Gly variant had a higher incidence of POAF following cardiac surgery compared to patients with the wild homozygous genotype (Arg389Arg). In contrast, they discovered that the link between the Gly389Gly variation and the risk of postoperative AF was no longer apparent in a subgroup of patients receiving perioperative beta-blockers, demonstrating that the risk of POAF was modulated by beta-blockers [21]. Results of the current study were in agreement with the aforementioned study as we could not find an association between Arg389Gly polymorphism and POAF in patients receiving perioperative beta-blockers for POAF prophylaxis. Our data revealed that the frequency of the rs1801253 G-allele in Egyptians was 41% which is similar to Africans (43%), lower than Europeans (32%) but higher than Eastern Asians (21%), and Southern Asians (27%).

This study has some limitations. Our cohort study consisted only of Egyptians. Thus, replication of our study to include different ethnic populations is recommended. In addition, further studies will be required to identify other clinical or genetic factors that may affect the response to beta-blockers. Machine learning techniques could be applied in future studies to identify novel genetic variants giving further predictive insights.

The current study has concluded that genetic polymorphism in GRK5 gene has an impact on the response to prophylactic beta-blockers in patients undergoing CABG. The use of prophylactic beta-blockers in GRK5-Leu41 variant allele carriers who are undergoing CABG is of no benefit; hence, other prophylactic medications with different mechanistic bases could be of more benefit in this specific population.

## Conclusion

The current study identified 2 major risk factors: age and GRK5 Gln41Leu polymorphism that could independently predict the response to beta-blockers used for POAF prophylaxis in Egyptian patients undergoing CABG. Since around 10% of Egyptians and 30% of Africans carry GRK5-Leu41, personalization of prophylactic therapy could positively impact the outcomes of CABG surgery.

## Data Availability

For the purpose of individual and patient privacy, data is not shared publicly. Yet it can be shared anonymously upon need or request.
